# Human notochordal cell transcriptome unveils potential regulators of cell function in the developing intervertebral disc

**DOI:** 10.1038/s41598-018-31172-4

**Published:** 2018-08-27

**Authors:** Ricardo Rodrigues-Pinto, Lizzy Ward, Matthew Humphreys, Leo A. H. Zeef, Andrew Berry, Karen Piper Hanley, Neil Hanley, Stephen M. Richardson, Judith A. Hoyland

**Affiliations:** 1 Division of Cell Matrix Biology and Regenerative Medicine, School of Biological Sciences, Faculty of Biology, Medicine and Health, The University of Manchester, Stopford Building, Oxford Road, Manchester, M13 9PT United Kingdom; 2 Spinal Unit, Department of Orthopaedics, Centro Hospitalar do Porto – Hospital de Santo António, Largo Prof. Abel Salazar, 4099-001 Porto, Portugal; 3 ICBAS - Instituto de Ciências Biomédicas Abel Salazar, Rua de Jorge Viterbo Ferreira n° 228, 4050-313 Porto, Portugal; 4 Faculty of Biology, Medicine & Health, University of Manchester, Oxford Road, Manchester, M13 9PT UK; 5 Division of Diabetes, Endocrinology & Gastroenterology, School of Medical Sciences, Faculty of Biology, Medicine & Health, University of Manchester, Oxford Road, Manchester, M13 9PT UK; 6 Research & Innovation, Manchester University NHS Foundation Trust, Grafton Street, Manchester, M13 9WU UK; 7 NIHR Manchester Biomedical Research Centre, Central Manchester Foundation Trust, Manchester Academic Health Science Centre, Manchester, United Kingdom

## Abstract

The adult nucleus pulposus originates from the embryonic notochord, but loss of notochordal cells with skeletal maturity in humans is thought to contribute to the onset of intervertebral disc degeneration. Thus, defining the phenotype of human embryonic/fetal notochordal cells is essential for understanding their roles and for development of novel therapies. However, a detailed transcriptomic profiling of human notochordal cells has never been achieved. In this study, the notochord-specific marker CD24 was used to specifically label and isolate (using FACS) notochordal cells from human embryonic and fetal spines (7.5–14 weeks post-conception). Microarray analysis and qPCR validation identified CD24, STMN2, RTN1, PRPH, CXCL12, IGF1, MAP1B, ISL1, CLDN1 and THBS2 as notochord-specific markers. Expression of these markers was confirmed in nucleus pulposus cells from aged and degenerate discs. Ingenuity pathway analysis revealed molecules involved in inhibition of vascularisation (WISP2, Noggin and EDN2) and inflammation (IL1-RN) to be master regulators of notochordal genes. Importantly, this study has, for the first time, defined the human notochordal cell transcriptome and suggests inhibition of inflammation and vascularisation may be key roles for notochordal cells during intervertebral disc development. The molecules and pathways identified in this study have potential for use in developing strategies to retard/prevent disc degeneration, or regenerate tissue.

## Introduction

Degeneration of the intervertebral disc (IVD) is associated with the development of low back and neck pain^1^, which are highly debilitating symptoms affecting up to 80% of the world population^2^. While current conservative and surgical therapies are relatively effective in relieving pain short term, they are not devoid of complications^3,4^ and fail to inhibit the degenerative process or promote repair. As such there is a need to develop alternative therapies that target the underlying aberrant molecular and cell biology.

However, to enable the development of novel biological or cell-based therapies for disc degeneration it is essential to characterise the pathways and processes involved in IVD development, maturation and degeneration. While in the embryonic, fetal and juvenile human IVD the nucleus pulposus (NP) is populated by large vacuolated notochordal cells, the adult disc is populated by small non-vacuolated chondrocyte-like cells (reviewed in^5^). Through study of animal tissue, notochordal cells have been proposed to play a fundamental role in IVD homeostasis^6–9^ and their loss with maturity in humans has been suggested to contribute to onset of the degenerative process^10^.

Thus, understanding the phenotype of notochordal cells and their potential regulatory molecules will help identify factors important in maintaining healthy disc homeostasis which may be exploited in the development of novel biological/regenerative therapies. Furthermore, the identification of human notochord-specific markers will further our understanding of whether notochord-derived cells persist in the adult NP. However, while studies have been undertaken using animal models^11–18^, to date the human notochordal cell phenotype has not been characterised in detail and this lack of understanding of human notochordal cell phenotype and biology is a major limitation in the field.

In a pivotal study using human embryonic and fetal spines, we have recently shown that the developing NP is composed of large vacuolated notochordal cells and that keratin (KRT) 8, KRT18, KRT19 are uniquely expressed by notochordal cells at all spine levels investigated at all stages studied (Carnegie Stage 10 (equivalent to 3.5 weeks post-conception (WPC)) to 18 WPC), with CD24 also being uniquely expressed at all stages except 3.5 WPC^19^.The unique expression of these markers makes them suitable for use in identification and isolation of notochordal cells from human embryos and foetuses and specifically CD24, being a cell-surface marker, allows for the isolation of viable notochordal cells.

Thus the hypotheses for this study were that: (i) the human developing NP contains notochordal cells which can be isolated from their adjacent sclerotomal cells by the unique expression of CD24; (ii) isolation of human notochordal cells will allow a characterisation of their phenotype and regulatory networks, upstream regulators and downstream functions allowing a better understanding of their function and role in the developing IVD and in protecting the IVD from degeneration and; (iii) the human adult NP contains cells that express notochordal cell markers, suggesting a persistence of notochordal cells in the human adult NP. As such, the aims of this study were to: (i) isolate viable notochordal cells from surrounding sclerotomal tissues of the human fetal spines; (ii) characterise the transcriptome of human notochordal cells and their potential regulatory networks and pathways; and (iii) assess whether notochord-derived cells are present in the human adult NP.

## Results

### Separation of CD24^+^ and CD24^−^ spine cells and qPCR validation of cell separation

**Figure 1:**
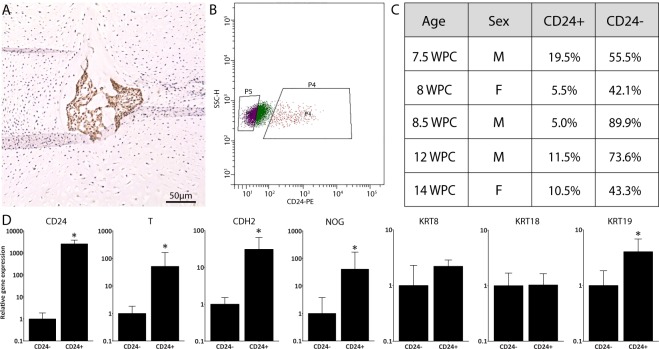
Isolation of viable human CD24^+^ (notochordal) cells and CD24^−^ (sclerotomal) cells from fetal human spines. (**A**) CD24 immunostaining of an 8 WPC human fetal spine showing notochord-specific staining. **(B)** FACS plot of the 12 WPC specimen used in the microarray analysis showing CD24-PE positivity against side scatter-height (SSC-H). P4 represents viable CD24^+^ events and P5 represents viable CD24^−^ events. **(C)** Proportion of viable CD24^+^ and CD24^−^ events sorted from each sample and predicted sex (based on transcriptome analysis) of each sample. **(D)** Relative gene expression of CD24, T, CDH2, Noggin, KRT8, KRT18 and KRT19 in CD24^+^ and CD24^−^ cells used in microarray analyses (N = 5). Gene expression was normalised to the reference gene GAPDH and then CD24^+^ cell expression was plotted relative to the expression in CD24^−^ cells and presented on a log scale. Error bars represent the standard error of the mean of the samples analysed. * represents p < 0.05.

Immunostaining of human developing spines confirmed discrete expression of CD24 within only large vacuolated notochordal cells of the developing NP, as previously described^19^ **(**Fig. 1A**)**. FACS analysis of human spine cells isolated from developing spines identified a small viable population (5.0–19.5%) of CD24^+^ cells within a larger viable population (42.1–89.9%) of CD24^−^ cells (Fig. 1B,C; Supplementary Figure 1). qPCR revealed significantly higher CD24, KRT19, CDH2, NOG and T gene expression in CD24^+^ than in CD24^−^ cells, confirming separation of CD24^+^ notochordal cells from CD24^−^ sclerotomal cells (Fig. 1D).

### Identification of CD24^+^ and CD24^−^ markers using microarrays

**Figure 2:**
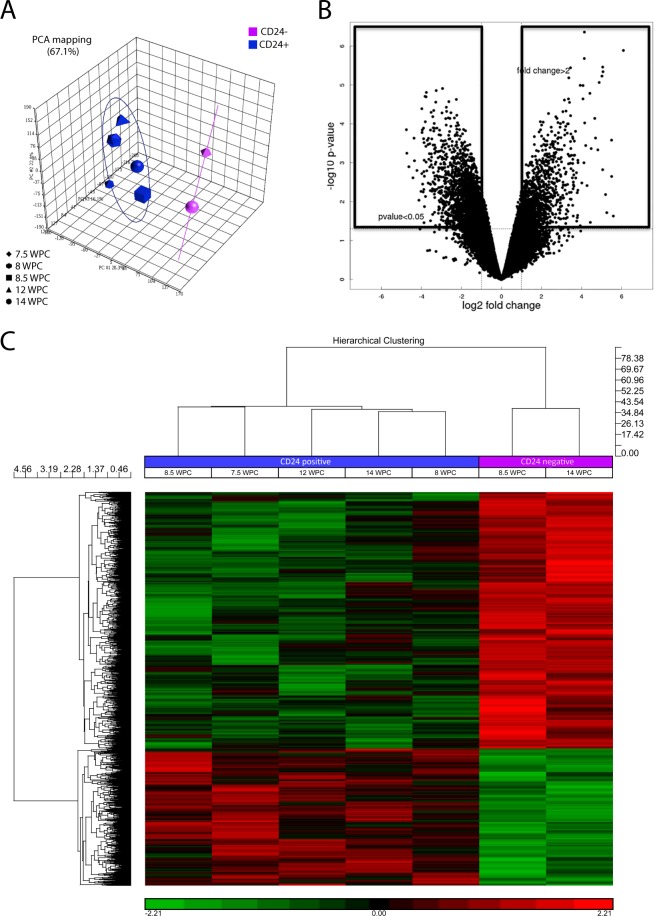
Bioinformatics analysis of microarray data. (**A)** Principal component analysis of the genes expressed by CD24^+^ (blue) and CD24^−^ (purple) cells. As CD24^+^ genes clustered in close proximity to each other and distant to CD24^−^ genes, this indicated a clear separation between two different cell types with a distinct gene expression. **(B)** Volcano plot depicting the differential gene expression signals between CD24^+^ and CD24^−^ genes. Only genes with a fold change <−2 or >2 and with a p-value < 0.05 were considered to be differentially expressed and were used for further analysis. **(C)** Hierarchical clustering of the genes in the 5 CD24^+^ (notochordal) and 2 CD24^−^ (sclerotomal) samples analysed. Each column represents one sample and each line represents one gene. Boxes in red indicate upregulation and boxes in green indicate downregulation. The dendogram identifies two main groups corresponding to CD24^+^ and CD24^−^ genes.

Three CD24^−^ samples failed to pass microarray quality control criteria and were excluded from further analysis. As such, microarray analysis was performed by comparing the expression of CD24^+^ samples from 5 and CD24^−^ samples from 2 specimens. PCA analysis demonstrated that the genes from CD24^+^ cells clustered together and away from those of CD24^−^ cells (Fig. 2A**)**. A differential expression test performed using a log ratio >2 or <2 and a p value ≤ 0.05 identified 884 up-regulated and 1460 down-regulated genes in CD24^+^ cells relative to CD24^−^ cells (Fig. 2B**)**. Hierarchical clustering showed segregation between CD24^+^ and CD24^−^ differentially expressed genes, with a similar gene expression profile between the cells from the 5 CD24^+^ samples, which differed substantially from that of the cells from the 2 CD24^−^ samples (Fig. 2C**)**. Together, these results indicate that the gene expression profile of CD24^+^ cells is distinct from that of CD24^−^ cells, demonstrating a clear separation between cell types.

Top notochordal markers were CD24, STMN2, RTN1, PRPH, CXCL12, IGF1, MAP1B, ISL1, CLDN1 and THBS2 and top sclerotomal markers were WISP3, CHST11, SERPINA3, CHAD, ZNF385B, LIF, PLA2G2A, PRSS33, FOSL1 and COL11A2 **(**Table 1**)**.

**Table 1:** Top (notochordal) and bottom (sclerotomal) differentially expressed genes between CD24^+^ and CD24^−^ cells identified from Affymetrix microarray analysis.

Symbol	Gene name	Fold Change	Log Ratio	p-value
**Notochordal Genes (Upregulated in CD24** ^**+**^ **Cells)**				
CD24	CD24 molecule	68.102	6.090	1.29 × 10^−06^
STMN2	stathmin-like 2	48.064	5.587	2.34 × 10^−02^
RTN1	reticulon 1	45.294	5.501	2.25 × 10^−03^
PRPH	peripherin	45.207	5.498	2.62 × 10^−04^
CXCL12	chemokine (C-X-C motif) ligand 12	33.187	5.053	1.51 × 10^−03^
IGF1	insulin-like growth factor 1 (somatomedin C)	28.073	4.811	4.65 × 10^−04^
MAP1B	microtubule-associated protein 1B	22.298	4.479	4.32 × 10^−04^
ISL1	ISL LIM homeobox 1	21.659	4.437	3.40 × 10^−02^
CLDN1	claudin 1	21.351	4.416	1.64 × 10^−05^
THBS2	thrombospondin 2	19.325	4.272	3.86 × 10^−03^
**Sclerotomal Genes (Downregulated in CD24** ^**+**^ **Cells)**				
WISP3	WNT1 inducible signaling pathway protein 3	−26.830	−4.746	1.41 × 10^−04^
CHST11	carbohydrate (chondroitin 4) sulfotransferase 11	−26.430	−4.724	5.69 × 10^−04^
SERPINA3	serpin peptidase inhibitor, clade A (alpha-1 antiproteinase, antitrypsin), member 3	−24.647	−4.623	1.27 × 10^−03^
CHAD	chondroadherin	−21.463	−4.424	2.44 × 10^−03^
ZNF385B	zinc finger protein 385B	−20.667	−4.369	2.33 × 10^−04^
LIF	leukemia inhibitory factor	−20.373	−4.349	8.86 × 10^−04^
PLA2G2A	phospholipase A2, group IIA (platelets, synovial fluid)	−18.677	−4.223	1.58 × 10^−03^
PRSS33	protease, serine, 33	−17.432	−4.124	9.16 × 10^−03^
FOSL1	FOS-like antigen 1	−16.776	−4.068	5.05 × 10^−02^
COL11A2	collagen, type XI, alpha 2	−16.233	−4.021	5.44 × 10^−03^

### Validation of identified markers using qPCR

**Figure 3:**
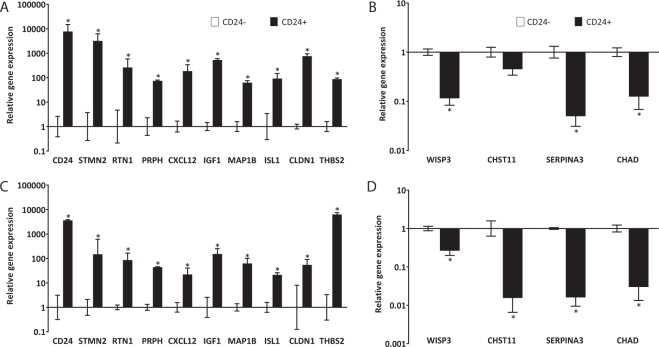
qPCR validation of differential gene expression. Relative expression of novel positive (**A**,**C**) and negative (**B**,**D**) notochordal marker genes in CD24^+^ (notochordal) cells relative to CD24^−^ (sclerotomal) cells isolated from the same human embryonic and fetal specimens used in the microarray analysis (**A**,**B**) and two additional specimens (9 and 11 WPC) (**C**,**D**). Gene expression was normalised to the reference gene GAPDH and the mean CD24^+^ cell expression was plotted relative to the mean expression in CD24^−^ cells and presented on a log scale. N = 5 for CD24^+^ and N = 2 for CD24^−^ samples in panels A and B; N = 2 for both CD24^+^ and CD24^−^ samples in panels C and D. Error bars represent the standard error of the mean of the samples analysed. * represents p < 0.05.

Differential expression of the top 10 CD24^+^ and top 4 CD24^−^ (bottom CD24^+^) differentially expressed genes was validated using qPCR, which revealed that all CD24^+^ markers CD24, STMN2, RTN1, PRPH, CXCL12, IGF1, MAP1B, ISL1, CLDN1 and THBS2 had significantly higher expression in CD24^+^ than in CD24^−^ cells (Fig. 3A). Similarly, the CD24^−^ markers WISP3, CHST11, SERPINA3 and CHAD had lower expression in CD24^+^ than in CD24^−^ cells (Fig. 3B); this difference was statistically significant for all markers except for CHST11.

To exclude the fact that concordant results between the microarray analysis and qPCR could be inherent to the biology of samples used in the microarrays, the expression of the same genes was analysed in RNA from CD24^+^ and CD24^−^ cell samples isolated from two additional specimens of similar ages (9 and 11 WPC). qPCR demonstrated that the expression patterns for top 10 CD24^+^ (Fig. 3C) and top 4 CD24^−^ markers (Fig. 3D) was similar to that seen in samples used in the microarray analysis (Fig. 3A,B respectively).

### Expression of notochordal markers in human adult NP samples

**Figure 4:**
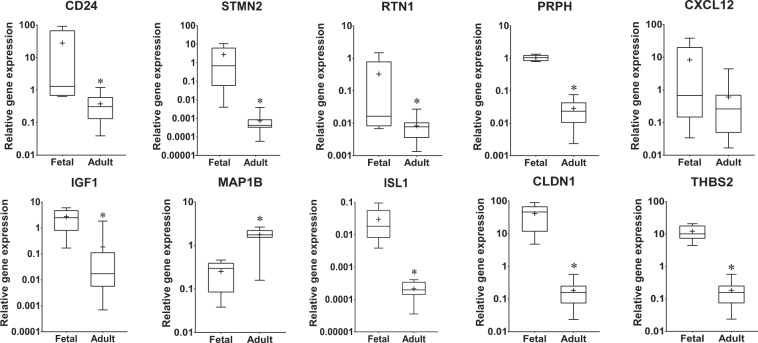
Comparison of expression of novel notochordal cell marker genes by human fetal notochordal cells and adult human NP cells. Box and whisker plots depicting the relative gene expression of CD24, STMN2, RTN1, PRPH, CXCL12. IGF1, MAP1B, ISL1 and CLDN1 in CD24+ (notochordal) cells (N = 5) and adult human NP cells (N = 17). Gene expression values were normalised to the reference gene GAPDH and plotted on a log scale. Graphs depict median, interquartile range and minimum/maximum values, with mean annotated as +. * represents p < 0.05.

To investigate the expression of notochordal cell markers in the adult NP and how expression differed from levels seen in fetal CD24+ notochordal cells, the expression of CD24, STMN2, RTN1, PRPH, CXCL12, IGF1, MAP1B, ISL1, CLDN1 and THBS2 was analysed in CD24^+^ sorted notochordal cells and NP cells isolated from adult tissue using qPCR. Importantly all notochordal markers were expressed in the NP of the cohort analysed (Fig. 4). Expression of CD24, STMN2, RTN1, PRPH, IGF1, MAP1B, ISL1, CLDN1 and THBS2 were all significantly lower in NP cells compared to notochordal cells, while expression of MAP1B was significantly increased in adult NP cells compared to fetal CD24^+^ notochordal cells.

Expression of the novel notochordal marker genes was also investigated in the adult NP cell cohort to determine whether their expression varied with different ages or stages of degeneration. No clear correlation was identified for the majority of the genes with ageing (Supplementary Figure 2) or with degeneration (Supplementary Figure 3); with only MAP1B showing a significant correlation with age or degeneration.

### Gene pathway analysis

To identify the networks, master regulators and biological functions of notochordal cells, dataset molecules (differentially expressed genes with −2 < FC < 2, p < 0.05) were subjected to IPA® analysis.

Within the top networks identified, those with highest known physiological relevance were: (i) connective tissue development and function (Supplementary Figure 4), (ii) nervous system development and function and (iii) development, skeletal and muscular disorders (Supplementary Table 3).

**Figure 5:**
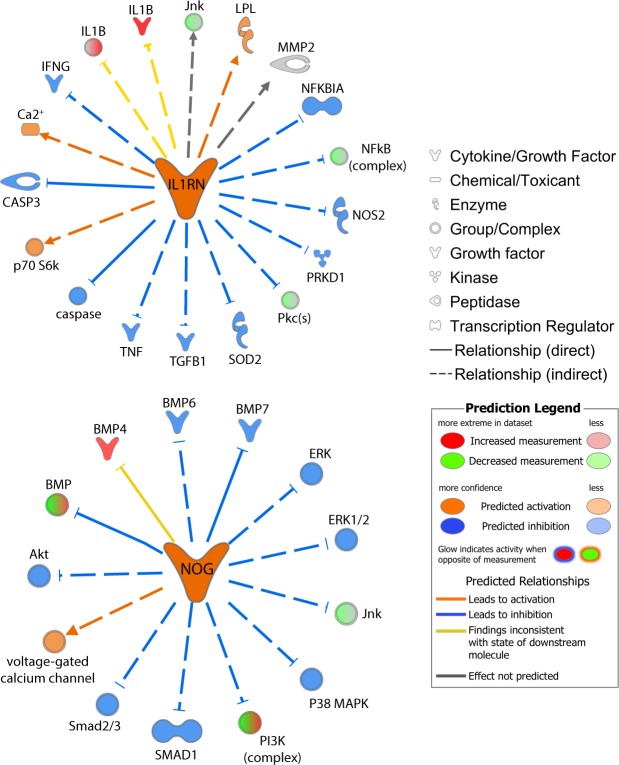
Predicted interactions between the growth factors (**A**) IL1RN, and (**B**) Noggin (NOG) and dataset genes generated through the use of IPA (QIAGEN Inc., www.qiagenbioinformatics.com/products/ingenuity-pathway-analysis/)^62^.

To identify which molecules regulate notochordal gene expression, master regulators were analysed. The list of master regulators was filtered to identify those which had a predictable activation state and that were either growth factors or cytokines (Table 2). These specific regulators were selected to better understand the specific microenvironment in which notochordal cells reside and which may thus control their function, and to identify potential factors which could drive stem/progenitor cell differentiation to notochordal cells. WNT1-inducible signalling pathway (WISP2), noggin and endothelin-2 (EDN2) were the growth factors predicted to be active master regulators of notochordal cell gene expression. IL-1 receptor antagonist (IL1RN), IL37 and sprouty-related EVH1 domain containing 2 (SPRED2) were the cytokines that were predicted to be active master regulators of notochordal cell gene expression. Noggin and IL-1RN were found to be upstream and may explain 13 and 19 up or downregulated dataset molecules respectively (Fig. 5). VEGFA and TGFβ1 were within the list of growth factors predicted to be inhibited and IL-2, TNF, IL-5 and IL-20 were within the list of cytokines predicted to be inhibited (Table 2).

**Table 2:** Master regulators of differentially expressed genes.

Master Regulator	Activation z-score	Log Ratio	p-value of overlap
**Growth factors predicted to be active**			
WISP2	3.253	−0.143	2.12E-24
NOG	3.103	−2.306	1.19E-14
EDN2	2.142	0.059	5.24E-08
**Cytokines predicted to be active**			
IL1RN	5.317	1.530	1.59E-22
IL37	4.730	0.068	2.05E-21
SPRED2	2.209	−0.888	3.77E-13
**Growth factors predicted to be inhibited**			
BDNF	−4.507	0.930	2.73E-22
NRG1	−4.415	1.170	1.64E-28
CLEC11A	−4.320	−0.498	8.39E-17
VEGFA	−3.967	−1.631	3.49E-24
IGF1	−3.768	4.811	1.32E-27
TGFB1	−3.762	−0.700	6.41E-17
EGF	−3.755	0.503	2.79E-11
GRP	−3.530	0.034	1.21E-22
JAG1	−3.516	1.344	9.11E-25
FGF1	−3.316	−0.513	1.60E-25
**Cytokines predicted to be inhibited**			
IL2	−5.607	0.510	5.71E-23
OSM	−5.088	−0.131	4.76E-20
CCL5	−4.899	0.207	2.36E-20
TNF	−4.807	0.425	4.55E-21
TNFSF13B	−4.741	0.236	5.89E-17
CXCL12	−4.434	5.053	8.25E-27
TRIP6	−4.426	−0.882	6.35E-12
VAV3	−4.330	1.178	8.42E-17
IL5	−4.202	0.046	2.08E-09
IL20	−4.122	−0.199	5.84E-20

Log2 ratio represents differential expression of the regulator in the dataset; activation z-score represents the activation state (inhibited if <−2 and active if >2), and p-values represent the likelihood of the regulator regulating the genes in the dataset not being due to chance.

To understand which diseases or functions may be affected by the genes in the dataset, top diseases and functions were analysed; only those with a predictable activated state (z score <2 for inhibition and >2 for activation) were considered. There were 503 dataset molecules associated with organism survival, and this function was predicted to be active. Within the top functions predicted to be inhibited downstream of the dataset molecules were development of blood vessels, vasculogenesis and angiogenesis **(**Table 3**)**.

**Table 3:** Top 20 diseases and functions associated with the genes in the dataset.

Categories	Diseases or functions annotation	p-Value	Predicted activation state	Activation z-score	Number of dataset molecules
Cellular Growth and Proliferation	proliferation of cells	1.39E-28	Decreased	−2.299	733
Cellular Movement	cell movement	2.35E-23	Decreased	−2.647	446
Cellular Movement	migration of cells	3.63E-22	Decreased	−2.479	405
Organismal Survival	organismal death	7.25E-21	Increased	3.156	503
Cancer	malignant neoplasm of abdomen	2.84E-18	Decreased	−2.558	812
Gene Expression	expression of RNA	1.47E-16	Decreased	−3.309	422
Cardiovascular System Development and Function	development of cardiovascular system	1.06E-15	Decreased	−2.709	238
Cardiovascular System Development and Function, Organismal Development	development of blood vessel	1.89E-15	Decreased	−2.704	194
Gene Expression	transcription	3.60E-15	Decreased	−3.354	379
Gene Expression	transcription of RNA	3.99E-15	Decreased	−3.294	373
Cardiovascular System Development and Function, Organismal Development	vasculogenesis	5.18E-15	Decreased	−2.652	176
Gene Expression	transcription of DNA	1.68E-13	Decreased	−2.011	298
Cellular Movement	cell movement of tumor cell lines	3.25E-13	Decreased	−2.189	183
Cellular Movement	migration of tumor cell lines	2.40E-12	Decreased	−2.058	151
Cardiovascular System Development and Function	angiogenesis	5.63E-12	Decreased	−2.624	160
Developmental Disorder, Skeletal and Muscular Disorders	congenital anomaly of musculoskeletal system	5.18E-11	Increased	2.298	147
Cardiovascular System Development and Function, Cellular Movement	cell movement of endothelial cells	6.18E-11	Decreased	−2.067	91
Cardiovascular System Development and Function, Tissue Development	development of cardiovascular tissue	6.31E-11	Decreased	−2.013	103
Cellular Growth and Proliferation	proliferation of epithelial cells	2.19E-10	Decreased	−2.259	102
Organismal Development	size of body	2.79E-10	Decreased	−2.293	193

Only diseases and functions with a predictable activation score (z-score <−2 and >2) and a p value < 0.05 were included.

## Discussion

While several studies have attempted to characterise the phenotype of notochordal cells in animal models^11–18^, results from these studies may not be directly translatable to human research as the NP cell (which derives from the notochordal cell) phenotype varies considerably between species^13,20,21^. Here, for the first time human notochordal cells were isolated and their phenotype characterised and validated at the transcriptome level. The findings presented confirm the robustness of our novel methodology to isolate human notochordal cells from sclerotomal cells using the recently identified specific human notochordal marker CD24^19^. Importantly our new methodology adds substantial novelty to the field as it can potentially be reproduced to isolate viable human notochord cells which can be used in *in vitro* experiments to better elucidate their function.

The CD24^+^ notochordal cells were shown to express a panel of known notochordal marker genes including T, CDH2, NOG, KRT8, 18 and 19, with the majority of genes showing significant differential expression between CD24^+^ notochordal cells and CD24^−^ sclerotomal cells. Interestingly, KRT8 and 18 did not show differential expression, conflicting with our earlier study demonstrating notochord-specific expression at protein level at the fetal stages studied here^19^. Regulation of keratin expression in other tissues, such as skin, has been shown to be complex and includes post-transcriptional regulation^22^. Such post-transcriptional regulation can vary according to cell type, local environmental factors, response to injury and age, and in the IVD differences between keratin gene and protein expression have been described with ageing^23^. Similar post-transcriptional regulation mechanisms may occur developmentally explaining differences observed between expression of keratins at gene and protein level in the human fetal notochordal and sclerotomal cells studied here.

Significantly, the notochordal markers identified here provide the first transcriptomic characterisation of human notochordal cells, and highlight genes that are relevant to the IVD physiology and homeostasis, some of which have already been proposed as biomarkers of a healthy IVD. Further investigations into the role of these genes in the adult normal and degenerate IVD may highlight their relevance in this tissue. Interestingly, and confirming the importance of analysing human tissue, although some of these markers have been linked to functions that may be relevant to their role in the IVD, from all the markers identified in this analysis, only CD24 had previously been associated with the notochord or with the notochordal rich NP^24^.

From the top notochordal cell markers identified here only IGF-1, CLDN1 and THBS2 have previously been associated with the IVD. IGF-1 has been suggested to play a fundamental role in rat, bovine and human NP cell proliferation, anabolism and homeostasis^25–28^ and IGF-1, along with other family members, have recently been identified in a transcriptomic profiling of mouse notochordal cells^15^. CLDN1 has also been previously identified in the IVD^29^. CLDN1 is involved in cell polarity in epithelial cells and its expression by notochordal cells may be explained by the fact that these cells also display epithelioid characteristics such as cell polarity and cell-cell contact^30^, which are lost with skeletal maturity. THBS2 is an IVD extracellular matrix protein regulating the levels of the catabolic proteins MMP2 and MMP9^31,32^. It has been hypothesised to be important for the maintenance of the avascular state of the human and sand rat IVD^33^ and the THBS2 rs9406328 polymorphism is associated with disc herniation^34^. THBS2 expression by notochordal cells further emphasises the importance of these cells in maintaining IVD homeostasis. While the other top markers identified from our study have been linked with functions such as embryogenesis, nervous system development, epithelial cell proliferation, cell adhesion and insulin regulation, it is unclear if these genes perform similar functions in human spine development.

Importantly all the top notochordal markers were expressed to different degrees in the adult NP and no correlation was identified with ageing or histological grade of degeneration. This extends our recently published data which demonstrated that the gene and protein expression of known notochordal markers did not vary with ageing or degeneration across a wide range of adult NP tissues^35^. The current data adds further evidence to the theory that notochordally-derived cells are indeed present in the adult human NP, as has been previously suggested^13,18,35,36^.

It has been suggested that notochordal cells may exert their protective and anabolic role due to soluble factors secreted by them^37,38^, particularly inflammatory mediators, anabolic factors such as CTGF and TGFβ1, and anti-angiogenic factors^9,39^ and this has been shown *in vitro* to be potentiated in hypoxic cultures^40^. However, these findings all derive from studies in animals and may not be directly translatable to human biology. As such, in this study, IPA was utilised to identify biological networks, master regulators and potential downstream functions of human notochordal cells.

The top biological networks associated with notochordal cells were “connective tissue development and function” and “nervous system development and function” possibly reflecting two of the main functions of the developing notochord: providing structural support and induction of the development of the neural tube^41,42^. Furthermore, notochordal signalling (particularly through Shh, Wnt and BMP) is fundamental for neural tube patterning, differentiation and development^41,43–45^.

When analysing master regulators of notochordal cell genes, three “growth factors” were predicted to be active: WISP2, NOG and EDN2. WISP2 (CCN5) is a member of the CTGF/CCN family of secreted, extracellular matrix-associated proteins^46^ that has been shown to be an inhibitor of angiogenesis^47^. Furthermore, this molecule has recently been identified in non-chondrodystrophic dog notochordal cell conditioned medium (together with TGFbeta1 and CTGF) and proposed to be important for the homeostatic regulation of the healthy NP^39^. Noggin is a BMP antagonist that is produced by the notochord and that is involved in somite patterning^48^ together with maintenance of an avascular state in the embryo’s midline. It has been previously shown that Noggin, together with chondroitin sulphate, are notochordal soluble factors responsible for inhibiting endothelial cell invasion and blood vessel formation in the IVD^49^. More recently, in a transcriptomic study of mouse notochord-derived cells, Peck and colleagues identified that expression of Noggin was significantly higher at embryonic stage 12.5 than at birth (P0) suggesting its role in early spine development^15^. While no studies have linked EDN2 to notochordal cell biology, it has been identified as a potent angiogenic inhibitor in the developing retina^50^. These three master regulators of notochordal cells have a common aspect, which is relevant to notochordal cell biology and, possibly to the notochordal cell role in protecting against IVD degeneration, i.e. they all inhibit angiogenesis. Interestingly, VEGFA was among the growth factor master regulators predicted to be inhibited, again suggesting that notochordal cell function is widely regulated by anti-angiogenic mechanisms.

Further highlighting the relevance of inhibition of angiogenesis as a key role of notochordal cells, IPA analysis of downstream functions, i.e. functions regulated by notochordal cells, identified several angiogenic functions (development of blood vessels, vasculogenesis and angiogenesis) and all were predicted to be inhibited downstream of notochordal cells. This anti-angiogenic function is key to notochordal cell biology, as one of the main functions attributed to the notochord during development is the maintenance of an avascular midline region in the embryonic tissue^51^. The negative regulatory function has been suggested to be induced by Noggin and Chordin (BMP antagonists) suppression of endothelial cell differentiation and maturation^49,52^. This avascular, and also aneural, state of the midline is seen in the fully matured healthy IVD. Conversely, degeneration of the IVD coincides with the migration of neo-nerve and -vessels to the IVD^1^. Crucially these data suggest that notochordal cells are responsible for inhibition of angiogenesis in the developing and, possibly, in the non-degenerate NP and that, with notochordal cell disappearance or loss of function, the loss of this inhibition may be important in the genesis of IVD degeneration.

Within the master regulators of notochordal cell gene expression, the pro-inflammatory cytokines IL-2 TNF, TNFSF13B, IL-5 and IL-20 were predicted to be inhibited and IL-1RN was predicted to be activate upstream of notochordal cell genes. The role of pro-inflammatory cytokines in IVD degeneration has been extensively studied, with their expression being upregulated in degenerated discs^53,54^ and an imbalance between IL-1ß and its inhibitor (IL-1RN) has been shown to be involved in the pathogenesis of IVD degeneration with the authors proposing IL-1RN as a potential therapy for disc degeneration^53,55^. Further emphasising the importance of IL-1RN in maintaining a healthy IVD, Phillips and colleagues^56^ have shown that IL-1RN null mice display typical features of IVD degeneration. Thus, since the loss of IL-1RN may be one of the key drivers of IVD degeneration, it is possible that the loss of this notochordal-driven anti-inflammatory role is involved in the protective role notochordal cells exert in the IVD.

To conclude this is the first study to isolate and phenotype human notochordal cells. Significantly this provides fundamental tools to isolate viable human notochordal cells for future culture studies and phenotypic markers to define the phenotype of native/cultured notochordal cells or differentiated stem/progenitor cells. Importantly, this study also supports our recent work demonstrating that sub-populations of notochordal cells persist in the adult NP^35^; however, the role of these notochord-derived cells in the adult NP remains to be elucidated. Finally, this seminal study unveils the networks, regulators and downstream functions of human notochordal cells and identifies inhibition of inflammation and of angiogenesis among the most relevant notochordal functions during IVD development and maturation. We postulate that strategies aimed at inhibiting inflammation and angiogenesis, or in elucidating the roles of master regulators in the developing and adult IVD, should be investigated as their regulation can potentially retard or even prevent IVD degeneration.

## Methods

### Embryonic and fetal samples

#### Sample acquisition and processing

Embryonic and fetal samples (n = 7) were obtained with ethical approval from the local research ethics committee (Manchester Royal Infirmary, Ref. No: 08/H1010/28 Early Pregnancy Tissue Collection) and with fully informed written consent following medical or surgical pregnancy termination. All experimental protocols were performed in accordance with National Research Ethics Service and University of Manchester guidelines and regulations. Embryonic staging was performed according to the Carnegie classification^57^ and converted to WPC. Fetal staging was estimated by hand and foot length measurements and spine development macroscopically resembled previously described morphology^19^. Samples were processed within 2–4 hours of acquisition and the whole spine containing the vertebrae and IVDs was dissected from its adjacent tissues under sterile conditions, using microsurgical instruments and a stereomicroscope (Stem 2000, Carl Zeiss®), as previously described^19^. A 5 µm section of formalin-fixed paraffin embedded tissue from fetal samples at the WPC stages used for dissection were subjected to CD24 immunostaining as previously described^19^. Sex of samples was determined retrospectively through analysis of expression levels of a panel of previously identified female (XIST) and male (KDM5D, DDX3Y and RPS4Y1) transcript biomarkers in the microarray datasets^58^ (Supplementary Figure 5).

#### Isolation of notochordal and sclerotomal cells

Following dissection, spines were cut into small fragments and digested for 2 hours in medium (alpha-MEM (Sigma-Aldrich®, M4526)) containing 1% (v/v) antibiotic/ antimycotic solution and 0.1% (w/v) type II collagenase (Gibco®, 17101-015) in an orbital shaker at 37 °C; cell clusters were subsequently dissociated by incubation for 10 minutes at 37 °C in cell dissociation solution (Sigma®, C1419). Cells were washed twice in FACS buffer (0.5% BSA in 2 mM EDTA (Sigma-Aldrich®, T4174) in PBS, sieved through a 40 µm cell filter, and labelled with 0.3 μM Draq7® and 0.5 μg/mL of PE-conjugated anti-CD24 antibody (Beckman Coulter®, Cat: PN IM1428U) in FACS buffer for 10 minutes in the dark, at 4 °C. Samples were then washed FACS buffer, re-suspended in ice-cold PBS and immediately used for FACS (FACS Aria II; BD Biosciences). An isotype control (0.2 μg/mL IgG1, BD Pharminogen®, 550617) and Draq7® viability dye (0.3 μM) were used to establish gates for viable CD24^+^ and CD24^−^ cells. Viable CD24^+^ and CD24^−^ cells were sorted separately into microcentrifuge tubes containing 350 μL of RLT lysis buffer (RNeasy micro plus kit, Qiagen®) and RNA extracted immediately. No differences were detected in the forward and side scatter distribution of CD24^+^ and CD24^−^ events within each sample.

#### RNA extraction and amplification

RNA from embryonic and fetal spines was obtained using the RNeasy micro plus kit (Qiagen®, 74034). The following changes were made to the manufacturer’s protocol: (i) cell lysate was warmed to 37 °C before homogenisation; (ii) to elute the RNA from the column, water was warmed to 60 °C; and (iii) to allow for higher RNA recovery, the final eluent was re-pipetted through the column.

Due to the small amount of RNA obtained (inherent to the small specimen size), RNA was amplified to Spia® (Single Primer Isothermal Amplification) cDNA using the Ovation Pico WTA v2 kit (CAT NuGen Technologies®, 3302), purified using the RNeasy MinElute kit (Qiagen®, 74204). All steps were performed according to manufacturer’s instructions. RNA integrity was analysed using the Agilent 2100 Bioanalyser (Agilent Technologies®). Only high quality RNA (RNA integrity number >7) was used for microarrays.

#### Validation of the cell separation method

Before undertaking microarray analysis and to confirm that the sorted cells were notochordal and sclerotomal, real-time quantitative PCR (qPCR) for the genes CD24, CDH2, T, KRT8, KRT18, KRT19, Noggin was performed using TaqMan Universal PCR Master Mix (Applied Biosystems®, 4304437) and gene expression levels compared between CD24^+^ and CD24^−^ viable sorted cells, as previously described^13,20^.

#### cDNA microarrays

RNA was fragmented and labelled using the Encore Biotin Module (NuGen Technologies®, 4200), according to manufacturer protocol.

Four micrograms of SPIA® cDNA from each sample was hybridized to the GeneChip human genome U133 Plus 2.0 array (Affymetrix®, 900466), according to manufacturer’s instructions. Technical quality control was performed with dChip software^59^; an array was considered to be an outlier when more than 5% of the probe sets for that array were judged as outliers by the dChip outlier detection algorithm. Background correction, quantile normalization, and gene expression analysis were performed using the robust multiarray average (RMA) analysis in the Bioconductor software package^60^. Differential expression analysis was performed using routine analytical methods (Smyth, 2004). Lists of differentially expressed genes were controlled for false discovery rate (FDR) errors using the QValue method^61^.

#### Microarray analysis

Differentially expressed genes were defined as those with an FDR-corrected *P* value (called the q value) of ≤0.05 and a minimum normalized expression level of ≥2 fold. CD24^+^ marker genes were those with the highest differential expression between CD24^+^ and CD24^−^ cells and CD24^−^ marker genes were those with the highest differential expression between CD24^−^ and CD24^+^ cells.

Hierarchical clustering, principal component analysis (PCA) and volcano plots were analysed using Partek Genomics Suite (Partek Inc®, St. Louis, MO, USA).

Ingenuity® Pathway Analysis Software (IPA) (QIAGEN Inc., www.qiagenbioinformatics.com/products/ingenuity-pathway-analysis/)^62^ was used to identify networks, master regulators and biological functions of differentially expressed genes. p-values were calculated using the right-tailed Fisher Exact Test; Benjamini-Hochberg test was used for p-value multiple testing correction.

IPA analyses performed were: IPA Network Analysis, IPA Functions and IPA Master Regulators. IPA Network Analysis identifies regulatory networks (protein-protein interactions) between genes in a dataset to help understand how those genes are biologically related. IPA Functions analyses biological functions and disease processes relevant to the dataset genes and predicts their activation state (active or inhibited), which is given by the z-score. z-scores greater than 2 (active) or smaller than −2 (inhibited) were considered significant. Master regulators are molecules that can affect the expression of regulators of the molecules in the dataset and can also be predicted to be active (positive master regulators) or inhibited (negative master regulators) and this information is given by the z score. The list of regulators was filtered to identify those with a predictable activation state (z-scores greater than 2 or smaller than −2) and that were either cytokines or growth factors.

#### qPCR validation of differentially expressed genes

After performing the microarrays, expression of the newly identified differentially expressed genes was analysed in CD24^+^ and CD24^−^ sorted cells using SYBR green^63^. In addition to confirming differential expression in samples used in the microarray analysis, expression was also assessed in CD24^+^ and CD24^−^ sorted cells from two additional developing spines (9 and 11 WPC). Pre-optimised SYBR green primers were purchased from PrimerDesign (Southampton, UK). The expression of each gene was normalised to the most stable reference gene (Glyceraldehyde 3-phosphate dehydrogenase (GAPDH)) and data was analysed using the previously described 2^−ΔCt^ and 2^−ΔΔCt^ methods^64,65^. PCR primers are detailed in Supplementary Table 1.

#### Analysis of Novel Notochordal Cell Gene Expression in Adult NP samples

Adult IVD tissue was obtained at the time of surgery from 17 patients with disc degeneration (diagnosed by magnetic resonance imaging) who underwent disc replacement or spinal fusion (Supplementary Table 2). Written informed consent was obtained from all patients and the study was approved by the local research ethics committee. NP tissues were dissected from surgical samples, grade of degeneration classified histologically using a previously published grading system^66^ and cells were extracted as previously reported^67^. RNA was extracted using TRIzol and reverse transcribed to cDNA using a high capacity cDNA reverse transcription kit with RNase inhibitor (Thermo Life Technologies), as previously described^13,20^, then gene expression analysed as described above.

#### Statistical analysis

Statistical analysis of qPCR data was performed with GraphPad InStat® software, with the Mann-Whitney U test being used to compare means and the Pearson test to correlate gene expression values with degree of degeneration. p-values less than 0.05 were considered significant.

## Electronic supplementary material

Supplementary information

## Data Availability

The datasets generated during and analysed during the current study are available from Array Express (https://www.ebi.ac.uk/arrayexpress/; accession number E-MTAB-6868).
