# Supplementary material for: Human notochordal cell transcriptome unveils potential regulators of cell function in the developing intervertebral disc
Source: Sci Rep. 2018 Aug 27;8:12866. doi: 10.1038/s41598-018-31172-4 (PMC6110784; doi:10.1038/s41598-018-31172-4)
Supplement: Supplementary file 1 — Supplementary information [file 41598_2018_31172_MOESM1_ESM.pdf]

Human notochordal cell transcriptome unveils potential regulators of cell function in the developing intervertebral disc

SUPPLEMENTARY INFORMATION

Ricardo Rodrigues-Pinto[1, 2, 3], MD PhD, FEBOT; Lizzy Ward[1], PhD; Matthew Humphreys[1] BSc, MSc; Leo A.H. Zeef[4], MSc, PhD; Andrew Berry[5], PhD; Karen Piper Hanley[5], PhD; Neil Hanley[5,6,7] PhD; Stephen M. Richardson[1*], BSc PhD; Judith A. Hoyland[1,7*], BSc PhD

1. Division of Cell Matrix Biology and Regenerative Medicine, School of Biological Sciences, Faculty of Biology, Medicine and Health, The University of Manchester, Stopford Building, Oxford Road, Manchester, M13 9PT, United Kingdom
2. Spinal Unit, Department of Orthopaedics, Centro Hospitalar do Porto – Hospital de Santo António, Largo Prof. Abel Salazar, 4099-001 Porto, Portugal
3. ICBAS - Instituto de Ciências Biomédicas Abel Salazar, Rua de Jorge Viterbo Ferreira nº 228, 4050-313 Porto, Portugal
4. Faculty of Biology, Medicine & Health, University of Manchester, Oxford Road, Manchester M13 9PT, UK
5. Division of Diabetes, Endocrinology & Gastroenterology, School of Medical Sciences, Faculty of Biology, Medicine & Health, University of Manchester, Oxford Road, Manchester M13 9PT, UK
6. Research & Innovation, Manchester University NHS Foundation Trust, Grafton Street, Manchester M13 9WU, UK
7. NIHR Manchester Biomedical Research Centre, Central Manchester Foundation Trust, Manchester Academic Health Science Centre, Manchester, United Kingdom

Supplementary Figure 1

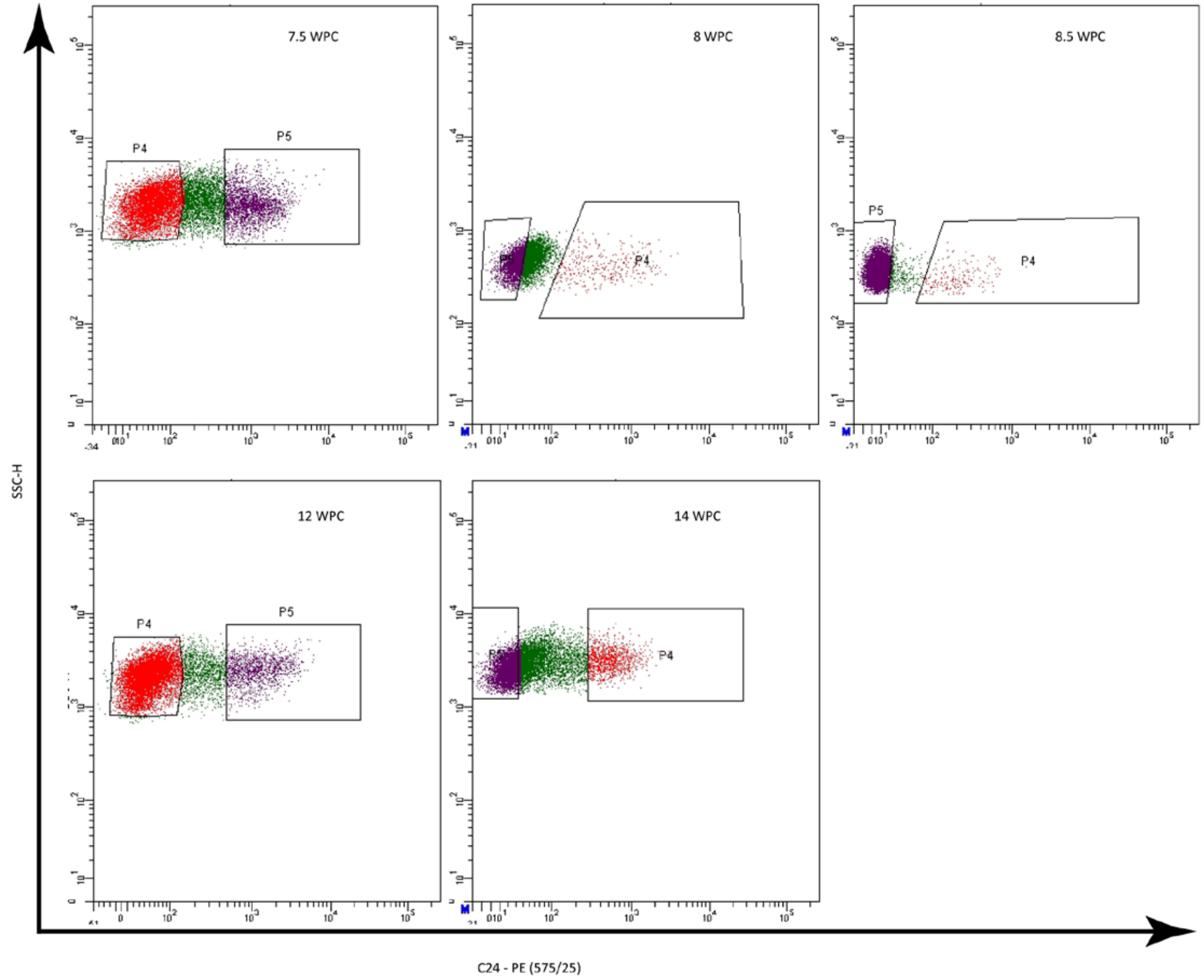

Supplementary figure 1. FACS plots of the samples used in the microarray analysis. Data plotted as CD24-PE positivity versus side scatter-height (SSC-H). P4 represents viable $CD24^+$ events and P5 represents viable $CD24^-$ events.

Supplementary Figure 2

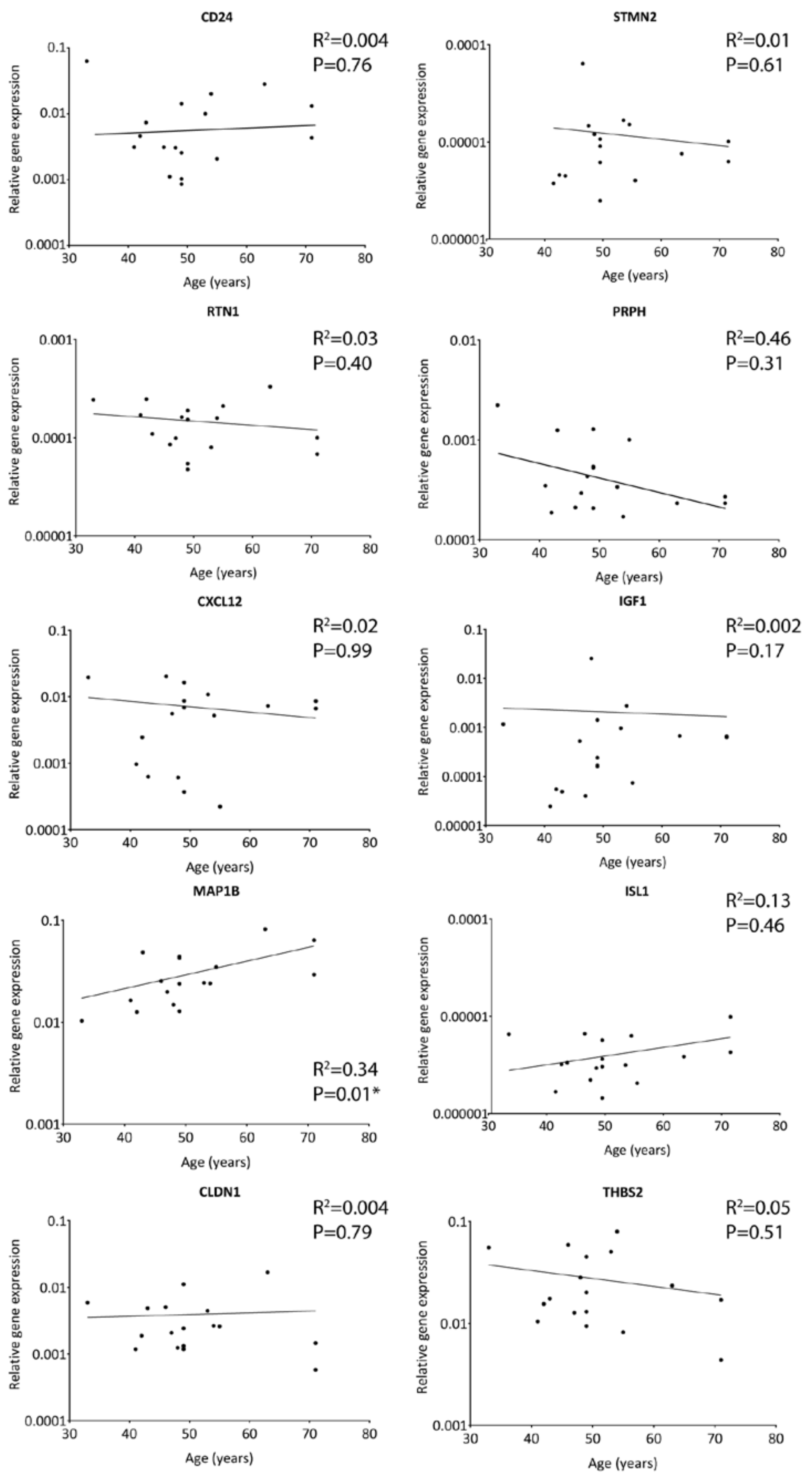

Supplementary figure 2. Scatter plots depicting the correlation between the expression of CD24, STMN2, RTN1, PRPH, CXCL12, IGF1, MAP1B, ISL1 and CLDN1 in the adult NP and patient age. Gene expression values were normalised to the reference gene GAPDH and plotted on a log scale vs patient age. Non-linear regression analysis was overlaid and R^2 and P value are presented for each gene.

Supplementary Figure 3

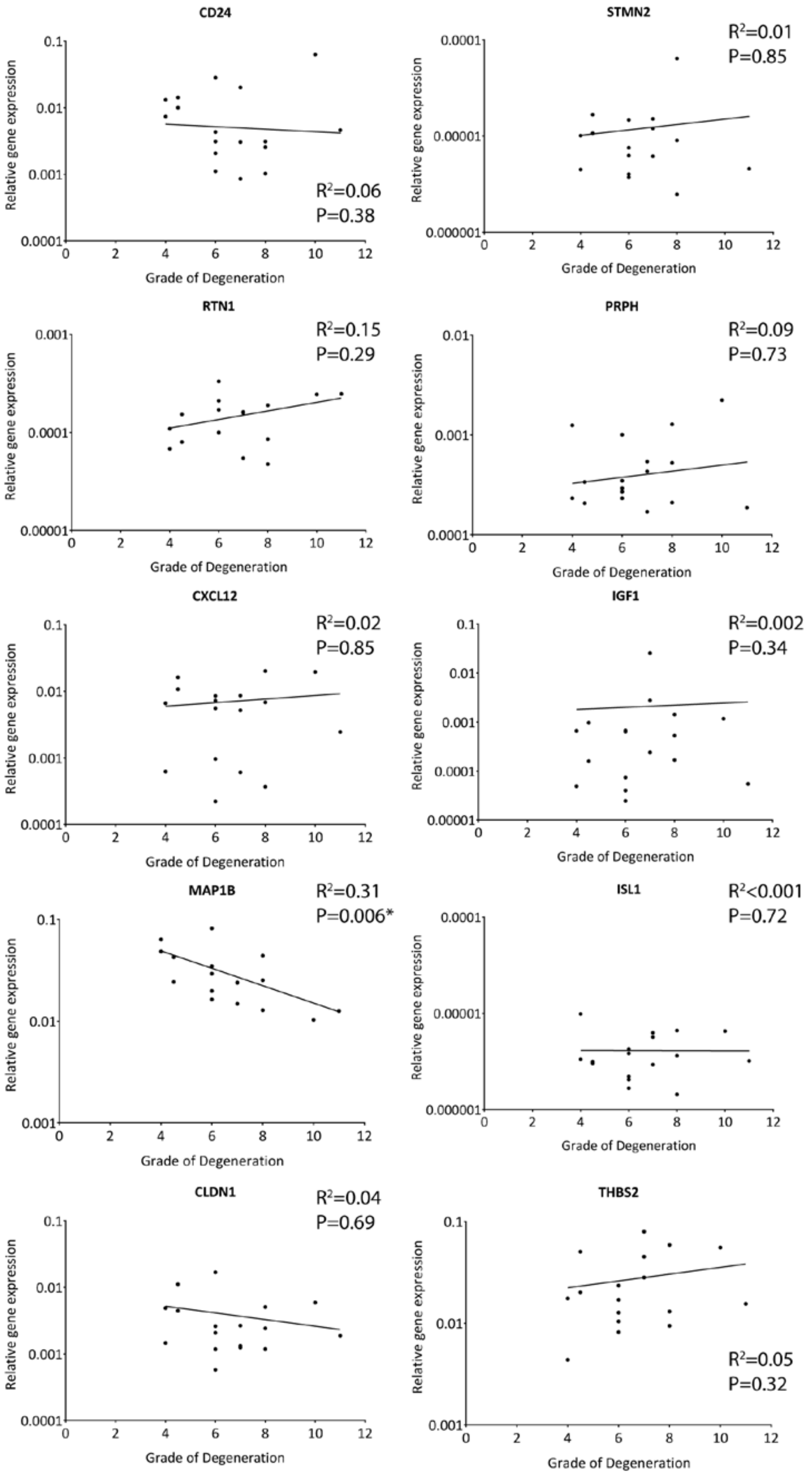

Supplementary figure 3. Scatter plots depicting the correlation between the expression of CD24, STMN2, RTN1, PRPH, CXCL12. IGF1, MAP1B, ISL1 and CLDN1 in the adult human NP and histological grade of degeneration. Gene expression values were normalised to the reference gene GAPDH and plotted on a log scale vs grade of degeneration. Non-linear regression analysis was overlaid and R^2 and P value are presented for each gene.

Supplementary Figure 4

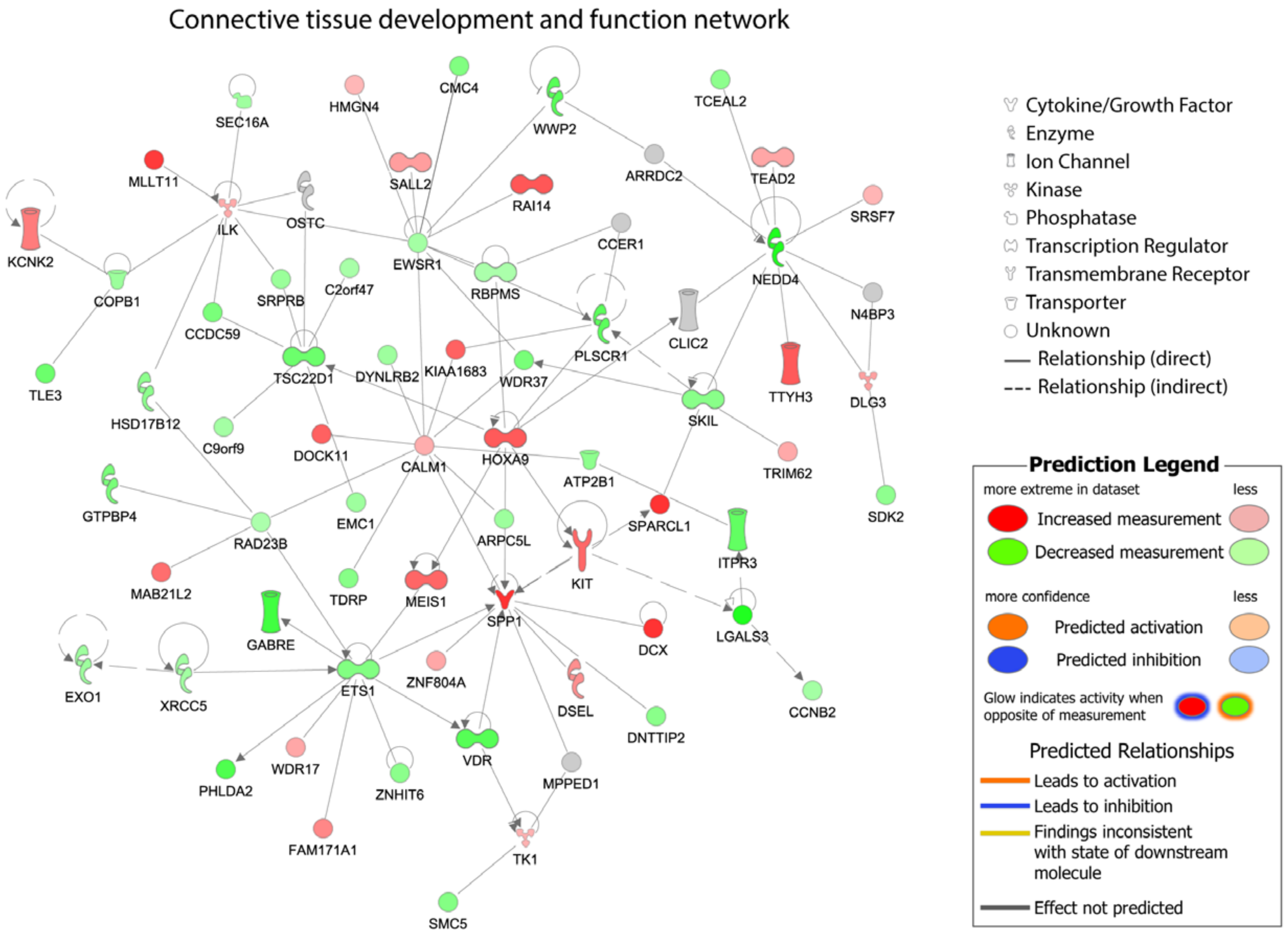

Supplementary figure 4. Sixty-four of the differentially expressed genes were related to "connective tissue development and function", which was the top scoring network. The network was generated through the use of IPA (QIAGEN Inc., www.qiagenbioinformatics.com/products/ingenuity-pathway-analysis)[1].

Supplementary Figure 5

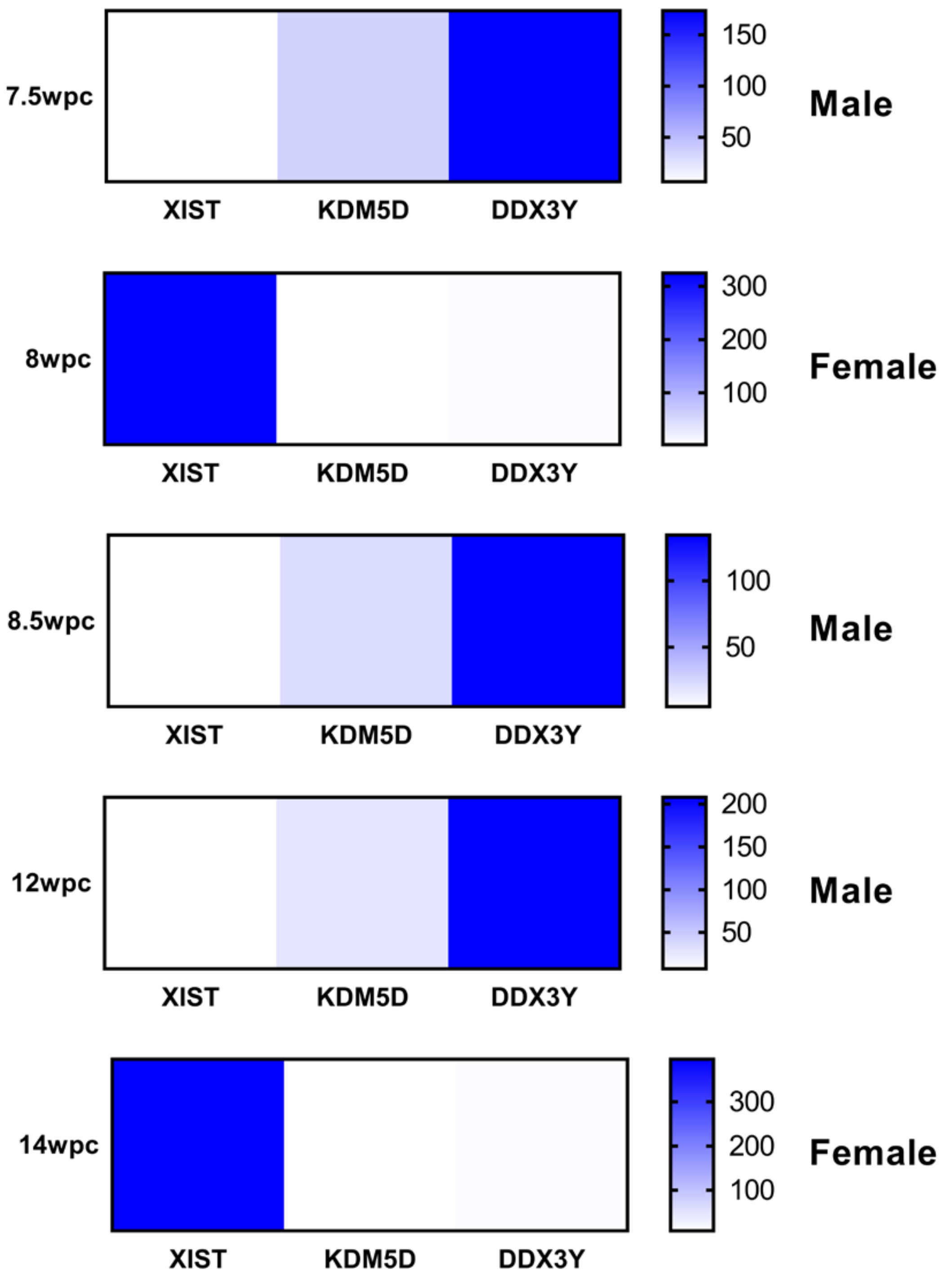

Supplementary figure 5. Heatmap summarising expression data for one female (XIST) and two male (KDM5D and DDX3Y) transcript biomarkers[2] identified in microarray data on CD24$^+$ notochordal cells form each sample. Values represent the mean of multiple probe values (XIST=6 probes; KDM5D=2 probes; DDX3Y=4 probes) with female samples predicted to have high XIST (blue) and low KDM5D and DDX3Y (white) and male samples predicted to have high KDM5D and DDX3Y and low XIST. Data identified 8 WPC and 14 WPC samples as female and 7.5 WPC, 8.5 WPC and 12 WPC samples as male.

Supplementary Table 1. Human oligonucleotide primers and probes used for qPCR analysis

Gene symbol	NCBI RefSeq	Forward primer	Reverse primer	Probe sequence	Optimal primer concentration (nM)
GAPDH	NM_001256799	CTCCTCTGACTTCAACAG	CGTTGTCATACCAGGAAA	CACCCACTCCTCCACCTTTGA	600
CD24	NM_001291737	GCTCCTACCCACGCAGATTTAT	CCTTGGTGGTGGCATTAGTTG	CCAGTGAAACAACAAC	900
STMN2	NM_001199214	AAGCCCCACGAACTTTAG	CGTGTTCCCTCTTCTCTG	N/A	900
RTN1	NM_021136	AATATCTGGGACTTGTGAGGACTC	TGTTACCACTCCAGACATTCCTG	N/A	900
PRPH	NM_006262	TGCCTCCTTAAATATAAAGACGAC	CCGGGTCTCAATGGTCTTG	N/A	900
CXCL12	NM_000609	GTGGTCGTGCTGGTCCTC	TCGGCATGGGCATCTGTAG	N/A	900
IGF1	NM_000618	GGTGGATGCTCTTCAGTTC	CTGCTGGAGCCATACCC	N/A	900
MAP1B	NM_005909	TGGGGAAGAGAAAGACAAGGA	GGGGCACAGCAGATGACT	N/A	900
ISL1	NM_002202	GCAGAGTGACATAGATCAGC	CTGTTAGGTGTATCTGGAAGTTG	N/A	900
CLDN1	NM_021101	TCCCAGAAGGCAGAGAGAGAAG	TCCCAGAAGGCAGAGAGAAG	N/A	900
THBS2	NM_003247	CACCAACGCCACCTACCA	CCGTCATTGTCATCGTCATCA	N/A	900
WISP3	NM_003880	TGCGACAGCAATATATTAAAGAC	GAGTACTTGAGCATCCAGAAAAG	N/A	900
CHST11	NM_001173982	CCTTTATCCTGGTCATCTTCTATTTC	GCTGGATTGGGTTGTAGAGTTC	N/A	900
SERPINA3	NM_001085	TCACAGGGGCCAGGAACC	GCAGAAAGGAGGGTGATTTTGAC	N/A	900
CHAD	NM_001267	AGCCCATGCCCCAACTC	TTTATAGAAATCTCAGGAAGAAATTGC	N/A	900

Supplementary Table 2. Details of the human adult NP samples used for gene expression analysis of identified notochordal markers. Grade refers to score out of 12 for grade of degeneration from a previously published histological grading system[3].

Anatomical region	Grade	Patient age (years)
C6/C7	4.5	49
C5/C6	4.5	53
C5/C6	4	43
C3/C4	4	71
C4/C5	6	71
C6/C7	6	47
C4/C5	6	55
C5/C6	6	63
C5/C6	6	41
C6/C7	7	49
L4/L5	7	54
C6/C7	7	48
C5/C6	8	49
L5/S1	8	46
C4/C5	8	49
L5/S1	10	33
L5/S1	11	42

Supplementary Table 3. High-scoring networks (Score >40) identified by IPA (QIAGEN Inc., www.qiagenbioinformatics.com/products/ingenuity-pathway-analysis)[1]. Each network is scored according to the number of focus molecules in the dataset and comprises a set of diseases and functions

ID	Score	Focus Molecules	Top Diseases and Functions
1	66	64	Connective tissue development and function, tissue development, cellular development
2	64	63	Post-translational modification, nervous system development and function, organ morphology
3	64	63	Cellular assembly and organization, nervous system development and function, cell morphology
4	63	64	Gene expression, cellular assembly and organization, cellular compromise
5	60	62	Reproductive system development and function, cancer, cellular development
6	60	61	Developmental disorder, skeletal and muscular disorders, cellular assembly and organization
7	58	61	Cell cycle, post-translational modification, cellular assembly and organization
8	57	60	Cellular movement, cellular development, cellular growth and proliferation
9	54	59	Embryonic development, organ development, organ morphology
10	52	59	Cellular assembly and organization, cell morphology, infectious disease
11	51	57	Behavior, nervous system development and function, embryonic development
12	51	56	Post-translational modification, hereditary disorder, neurological disease
13	49	55	Developmental disorder, hereditary disorder, organismal injury and abnormalities
14	49	55	Endocrine system development and function, molecular transport, protein synthesis
15	49	55	Cell-mediated immune response, cellular development, cellular function and maintenance
16	47	54	Cellular assembly and organization, nervous system development and function, cellular function and maintenance
17	44	52	Cell cycle, gene expression, cardiovascular disease
18	41	50	Lipid metabolism, small molecule biochemistry, molecular transport
19	41	50	Connective tissue disorders, dermatological diseases and conditions, gastrointestinal disease
20	39	49	Molecular transport, hematological disease, cellular movement
21	38	48	Gene expression, protein synthesis, RNA post-transcriptional modification
22	37	48	Cell signaling, cellular growth and proliferation, cellular movement
23	36	48	Developmental disorder, skeletal and muscular disorders, connective tissue disorders
24	36	47	Cellular assembly and organization, cellular movement, cell-to-cell signaling and interaction
25	35	47	Connective tissue disorders, DNA replication, recombination, and repair, gene expression

Supplementary information references

1 Kramer, A., Green, J., Pollard, J., Jr. & Tugendreich, S. Causal analysis approaches in Ingenuity Pathway Analysis. *Bioinformatics* **30**, 523-530, doi:10.1093/bioinformatics/btt703 (2014).

2 Staedtler, F. *et al.* Robust and tissue-independent gender-specific transcript biomarkers. *Biomarkers* **18**, 436-445, doi:10.3109/1354750X.2013.811538 (2013).

3 Sive, J. I. *et al.* Expression of chondrocyte markers by cells of normal and degenerate intervertebral discs. *Mol Pathol* **55**, 91-97 (2002).
